# Genetic insights into novel lysis suppression by phage CSP1 in *Escherichia coli*

**DOI:** 10.71150/jm.2501013

**Published:** 2025-04-29

**Authors:** Moosung Kim, Sangryeol Ryu

**Affiliations:** 1 Department of Food and Animal Biotechnology, College of Agriculture and Life Sciences, Seoul National University, Seoul 08826, Republic of Korea; 2 Department of Agricultural Biotechnology, College of Agriculture and Life Sciences, Seoul National University, Seoul 08826, Republic of Korea; 3 Research Institute of Agriculture and Life Sciences, College of Agriculture and Life Sciences, Seoul National University, Seoul 08826, Republic of Korea; 4 Center for Food and Bioconvergence, Seoul National University, Seoul 08826, Republic of Korea

**Keywords:** *Escherichia coli*, antiholin activity, lysis inhibition, mutation, virulent phage

## Abstract

Lysis inhibition (LIN) in bacteriophage is a strategy to maximize progeny production. A clear plaque-forming mutant, CSP1C, was isolated from the turbid plaque-forming CSP1 phage. CSP1C exhibited an adsorption rate and replication dynamics similar to CSP1. Approximately 90% of the phages were adsorbed to the host cell within 12 min, and both phages had a latent period of 25 min. Burst sizes were 171.42 ± 31.75 plaque-forming units (PFU) per infected cell for CSP1 and 168.94 ± 51.67 PFU per infected cell for CSP1C. Both phages caused comparable reductions in viable *E. coli* cell counts at a low multiplicity of infection (MOI). However, CSP1 infection did not reduce turbidity, suggesting a form of LIN distinct from the well-characterized LIN of T4 phage. Genomic analysis revealed that a 4,672-base pairs (bp) DNA region, encompassing part of the tail fiber gene, *CSP1_020*, along with three hypothetical genes, *CSP1_021*, *CSP1_022*, and part of *CSP1_023*, was deleted from CSP1 to make CSP1C. Complementation analysis in CSP1C identified *CSP1_020*, *CSP1_021*, and *CSP1_022* as a minimal gene set required for the lysis suppression in CSP1. Co-expression of these genes in *E. coli* with holin (*CSP1_092*) and endolysin (*CSP1_091*) resulted in lysis suppression. Lysis suppression was abolished by disrupting the proton motive force (PMF), supporting their potential role as antiholin. Additionally, CSP1_021 directly interacts with holin, suggesting that it may function as an antiholin. These findings identify new genetic factors involved in lysis suppression in CSP1, providing broader insights into phage strategies for modulating host cell lysis.

## Introduction

Bacteriophages (phages) are viruses that infect bacteria and lyse their host to release newly assembled virions. One of the well-characterized phage lysis systems is the holin-endolysin system. In phages that infect Gram-negative bacteria, the holin-endolysin system requires holin, endolysin, and spanin. These lysis proteins work together to disrupt the bacterial envelope at a precise time, ensuring maximum virions release (Cahill and Young, 2019; Young, 1992, 2014). They accumulate in the phage-infected cell without triggering premature lysis, as holin tightly regulates lysis timing (Mehner-Breitfeld et al., 2021; Schwarzkopf et al., 2024). Holins accumulate in the inner membrane and form pores, allowing endolysin to access the peptidoglycan layer. While the PMF is maintained, holin remains inactive and does not form pores. PMF collapse is a key trigger for pore formation, allowing endolysin to degrade the cell wall and initiate lysis at the optimal time (Guo et al., 2018; Hays and Seed, 2020). In temperate phages like Lambda, holin pores are formed through the process of oligomerization once they reach a critical threshold concentration. This pore formation subsequently leads to PMF collapse (Cahill et al., 2024; Cahill and Young, 2019). Spanins mediate the fusion of the inner and outer membranes, leading to phage release. This membrane fusion creates a localized fusion point. The internal turgor pressure causes catastrophic lysis, enabling efficient release of newly assembled phage particles (Kongari et al., 2018).

Some phages have evolved LIN to enhance the progeny production by delaying host cell lysis (Groman, 1965; Hays and Seed, 2020; Hvid and Mitarai, 2024; Lee et al., 2014; Wu et al., 2021). A notable example is LIN in phage T4, in which superinfection triggers LIN. LIN allows the phage to replicate continuously while preventing premature resource depletion (Chen and Young, 2016; Dressman and Drake, 1999; Krieger et al., 2020). This strategy provides a survival advantage, particularly in environments where host cells are scarce. By delaying lysis, phages maximize progeny production before the host is lysed (Abedon, 2019). In T4, LIN is regulated by antiholin proteins. For example, the RI antiholin protein binds to the C-terminal of T4 holin in the periplasm, where the C-terminal domain is exposed. This interaction prevents pore formation, delaying endolysin access to the bacterial cell wall (Mehner-Breitfeld et al., 2021; Tran et al., 2007). Additionally, the RIII antiholin protein functions as a secondary LIN modulator in the cytoplasm (Chen and Young, 2016). By binding on the N-terminal domain of holin, RIII protein cooperates with RI protein to reinforce holin inhibition. This effect is especially pronounced under stress conditions, such as superinfection, which extends the lysis inhibition period (Chen and Young, 2016; Schwarzkopf et al., 2024). T4 LIN results in synchronized lysis, maximizing progeny release once LIN is lifted (Abedon, 2019). This strategy allows T4 to optimize progeny production under favorable conditions, ensuring that a large number of phages released to enhance infection potential in the environment (Abedon, 2019).

CSP1 was previously isolated and characterized (Kim et al., 2024). Whole-genome sequencing identified CSP1 as a virulent phage belonging to the genus *Seuratvirus*. Although CSP1 forms turbid plaques, clear plaques were consistently observed during *E. coli* infections. We isolated a phage designated as CSP1C, which produces clear plaques and found that CSP1C is a deletion mutant of CSP1. Analysis of the deleted genes in CSP1 suggests their involvement in a LIN-like lysis suppression mechanism that regulates host cell lysis. Unlike T4, where LIN is triggered by superinfection and involves synchronized lysis for maximal progeny release, CSP1 employs a slow lysis process. This process is regulated by a distinct cluster of genes, *CSP1_020*, *CSP1_021*, and *CSP1_022*, even at a low MOI.

## Materials and Methods

### Bacterial strains and growth conditions

All plasmids and bacterial strains used in this study are listed in Tables S1 and S2. Bacterial strains were grown aerobically in Luria-Bertani (LB) medium at 37°C. When required, antibiotics were added at final concentrations of 100 µg/ml of carbenicillin (Cb), 50 µg/ml of kanamycin (Km), and 25 µg/ml of chloramphenicol (Cm).

### Phage purification

Phage purification was performed as previously described (Kim et al., 2024). Briefly, plaques with distinct morphologies were picked using sterilized pipette tips, resuspended in 1 ml of SM buffer [50 mM Tris-HCl (pH 7.5), 100 mM NaCl, 10 mM MgSO_4_], and homogenized. The homogenized suspension was filtered through a 0.22 µm pore-size filter (Millipore, Ireland) and subjected to plaque assays. To ensure phage purity, this process was repeated at least three times.

### Bioinformatics analysis

The phage DNA was extracted from 10^9^ PFU/ml of phage lysates as described by Sambrook et al. (1989). The extracted DNA was sequenced using the Illumina Miseq platform (Sanigen, Korea). Open reading frames (ORFs) were predicted using GenemarkS (Georgia Institute of Technology), FGENESB (softberry), and GLIMMER-3 (NCBI), and their functions were annotated using CLC Genomics Workbench 9.0 (QIAGEN Aarhus), incorporating NCBI BLAST (Basic Local Alignment Search Tool) and interproscan-5.13-52.0. Functional predictions of CSP1 genes were further refined using the MPI Bioinformatic Toolkit (Gabler et al., 2020). To identify mutation sites, sequence alignment between CSP1 and CSP1C was conducted using CLC Main Workbench v7.7.1 (QIAGEN Aarhus). Transmembrane domains of the proteins were predicted using TMHMM-2.0 (Hays and Seed, 2020; Krogh et al., 2001; Sonnhammer et al., 1998), and signal peptides were analyzed with SignalP 5.0 (Almagro Armenteros et al., 2019).

### Adsorption assay and one-step growth curve

The phage adsorption assay was performed as previously described with some modification (Kim et al., 2024). *E. coli* MG1655 (OD_600_ = 0.3–0.5) were mixed with phages at an MOI of 0.01 and incubated at 37°C. Samples were collected at 0, 3, 6, 9, and 12 min. The harvested samples were centrifuged (10,000 × g, 10 min, 4°C) and filtered through a 0.22 μm filter to remove bacterial cells and adsorbed phages. Unadsorbed free phages in the filtered lysate were quantified by plaque assay.

For the one-step growth curve, *E. coli* MG1655 (OD_600_ = 0.3–0.5) were centrifuged (6,000 × g, 10 min, 4°C) and washed with fresh LB broth. The bacterial pellet was resuspended, and phages were added at an MOI of 0.001. After 10 min of incubation at room temperature, unadsorbed phages were removed by centrifugation (6,000 × g, 10 min, 4°C) and discarding the supernatant. The pellet was resuspended in fresh LB medium and incubated aerobically at 37°C. Two sets of samples were collected every 5 min. The first set was directly utilized for plaque assays to analyze the latent period and burst size. The second set was treated with 1% (v/v) chloroform before plaque assay to release intracellular phage.

### TEM analysis

Purified CSP1 and CSP1C (10^10^ PFU/ml) were negatively stained with uranyl acetate. Phage-infected bacterial suspensions were also stained after collecting 2 h post-infection at an MOI of 0.1. The samples were adsorbed onto Formvar carbon-coated copper grids for 2 min and stained with 1% uranyl acetate for 2 min. Morphological details were observed using an Energy-filtered transmission electron microscope (EF-TEM, LEO 192 AB, Karl Zeiss, Germany) operating at 180 kV.

### Lytic activity analysis

Lysis activity of CSP1 and CSP1C was assessed by measuring absorbance at 600 nm (OD_600_) and bacterial concentration in phage-infected *E. coli* MG1655 cultures. Early exponential-phase *E. coli* MG1655 cultures (OD_600_ = 0.3–0.4) were infected with CSP1 or CSP1C at MOIs of 0.1, 0.01, and 0.001, and incubated aerobically at 37°C. Bacterial growth was monitored by measuring OD_600_ periodically using a SpectraMax i3 microplate reader (Molecular Devices). To evaluate reductions in viable cell, phages were added to *E. coli* MG1655 cultures (OD_600_ = 0.1–0.2) at an MOI of 0.1 and incubated aerobically at 37°C, and samples were collected. OD_600_ was measured, and samples were serially diluted and plated on LB agar to count viable cells.

Phage titers of CSP1 and CSP1C were measured hourly over 5 h. Exponentially growing *E. coli* MG1655 culture (OD_600_ = 0.1–0.2) were infected with CSP1 or CSP1C, and samples were collected at 1-h intervals. Each sample was treated with or without 1% chloroform to lyse unlysed cells, followed by centrifugation (10,000 × g, 10 min, 4°C) and filtration. Phage concentration in the filtered lysate was determined by plaquing assay.

### Statistical analysis

Statistical analyses were performed using GraphPad Prism 5. All experiments were conducted in triplicate. Phage particles increase over time was analyzed using one-way ANOVA, and comparisons of titer of CSP1C before and after chloroform treatment were evaluated using two-way ANOVA. Statistical significance was denoted by asterisks.

### Phage genome editing

Genome editing using the CRISPR-Cas9 system was performed as described by Hutinet et al. (2019) with modifications. A 20-bp spacer sequence targeting the desired site was designed (Table S3) and ligated into a BsaI-digested pCas9 plasmid. The recombinant plasmid was transformed into *E. coli* DH5α via heat shock. Repair plasmids were constructed using Gibson assembly with a pUC19 backbone. Repair templates lacking the target region were generated by amplifying 100-bp upstream and downstream sequences flanking the deletion site via PCR. These fragments were assembled with linearized pUC19 using a 2X Gibson assembly mix (NEB, USA) at 50°C for 1 h and transformed into *E. coli* DH5α by heat shock. The primer sequences for plasmids construction are listed in Table S4.

The constructed plasmids were introduced into *E. coli* MG1655 by electroporation. After overnight incubation, *E. coli* cells harboring both recombinant pCas9 and repair plasmid were subjected to a plaque assay to generate mutant phages. Individual plaques were picked and suspended in distilled water, followed by PCR screening to identify plaques containing mutant phages. Plaque suspensions confirmed to contain mutant phages were re-plated, and the plaque isolation process was repeated multiple times to eliminate the wild-type phage. The purified mutant phages were confirmed by PCR and sequencing to verify the mutation.

### Gene cloning and expression

The complementation analysis used three plasmid backbones: pACYC184, pBAD33, and pUHE21-2 *lacI^q^*. *CSP1_020*, *CSP1_020* to *CSP1_021*, *CSP1_020* to *CSP1_022*, and *CSP1_020* to *CSP1_023* were inserted into pACYC184. *CSP1_021* was inserted into pBAD33, and *CSP1_022* was inserted into pUHE21-2 *lacI^q^*. Genes were amplified using appropriate primers (Table S5) and inserted into the plasmid after BamHI/SalI digestion. Similarly, *CSP1_091* gene (endolysin) and *CSP1_092* gene (holin) were inserted into pBAD33 via BamHI/SalI sites using appropriate primers (Table S6). The recombinant plasmids were transformed into *E. coli* MG1655.

For determining antiholin activity, endolysin and holin were co-expressed in the pETDuet-1 vector (Meng et al., 2022; Wu et al., 2021; Zhang et al., 2022). The endolysin gene was inserted into multiple cloning site 1 using BamHI/SalI, and the holin gene was inserted into multiple cloning site 2 using XbaI/KpnI (Table S7). Additionally, *CSP1_020*, *CSP1_021*, and *CSP1_022* were cloned into pBAD33 for controlled expression, and the resulting plasmids were transformed into *E. coli* BL21 (DE3).

### Systematic complementation analysis

To identify the mutation responsible for clear plaque-forming phenotype of CSP1C, a plaquing assay was performed using CSP1Δ*CSP1_029* and CSP1Δ*CSP1_020*, *CSP1_021*, *CSP1_022*, and *CSP1_023* on *E. coli* MG1655.

To identify the specific gene responsible for lysis suppression, *E. coli* MG1655 harboring plasmids carrying different combinations of *CSP1_020*, *CSP1_021*, *CSP1_022*, and *CSP1_023* were infected with CSP1C. Genes expression from pBAD33 was induced with 0.2% L-arabinose, and genes expression from pUHE21-2 *lacI^q^* was induced with 0.01 mM IPTG. CSP1C was infected 2 h after induction at an MOI of 0.1, and growth inhibition was monitored by measuring OD_600_ periodically using a SpectraMax i3 microplate reader.

### Antiholin activity test

The antiholin activity of CSP1_020, CSP1_021, and CSP1_022 was evaluated in *E. coli* MG1655 and BL21 (DE3). In *E. coli* MG1655, endolysin or holin overexpression was induced by adding 0.02% L-arabinose to cells harboring pB91 or pB92 (Table S2). After 2 h post-induction, CSP1 or CSP1C was added at an MOI of 0.1, and OD_600_ was measured periodically using a SpectraMax i3 microplate reader (Molecular Devices).

In *E. coli* BL21 (DE3), cells harboring p12 and either pB (p12pB) or pB02 (p12pB02) were used for the assay (Table S2). In exponentially growing cultures (OD_600_ = 0.2–0.3), expression of holin and endolysin was induced with 0.2 mM IPTG, and expression of CSP1_020, CSP1_021, and CSP1_022 was induced with 0.2% L-arabinose. Cultures were incubated at 37°C, and OD_600_ was measured periodically using a SpectraMax i3 microplate reader (Molecular Devices). To assess PMF dependency, 2 mM DNP was added 1 h post-induction.

### Bacterial two-hybrid assay

A bacterial two-hybrid assay was performed to analyze interactions between holin and CSP1_020, CSP1_021, and CSP1_022. The vectors pKT25 and pUT18C were used for cloning, with pKT25-Zip and pUT18C-Zip as positive controls and empty vectors as negative controls (Karimova et al., 2001).

Each target gene (*CSP1_020*, *CSP1_021*, *CSP1_022*, and *CSP1_092*) was amplified using specific primers (Table S8) and digested with XbaI and KpnI. *CSP1_020*, *CSP1_021*, and *CSP1_022* were ligated into pKT25, while *CSP1_092* was ligated into pUT18C. The recombinant plasmids were transformed into *E. coli* BTH101 for the assay.

To detect interaction between holin and each of CSP1_020, CSP1_021, and CSP1_022, 40 µl of X-gal (20 mg/ml) and 100 μl of IPTG (100 mM) were spread onto LB agar plates containing Cb and Km. Two microliters of overnight *E. coli* BTH101 cultures harboring recombinant plasmids were spotted onto the prepared plates. After air drying at room temperature, the plates were incubated at 30°C for 20 h. Interaction was indicated by the development of blue coloration.

## Results and Discussion

### Phenotypic comparison of CSP1 and CSP1C

**Fig. 1.**
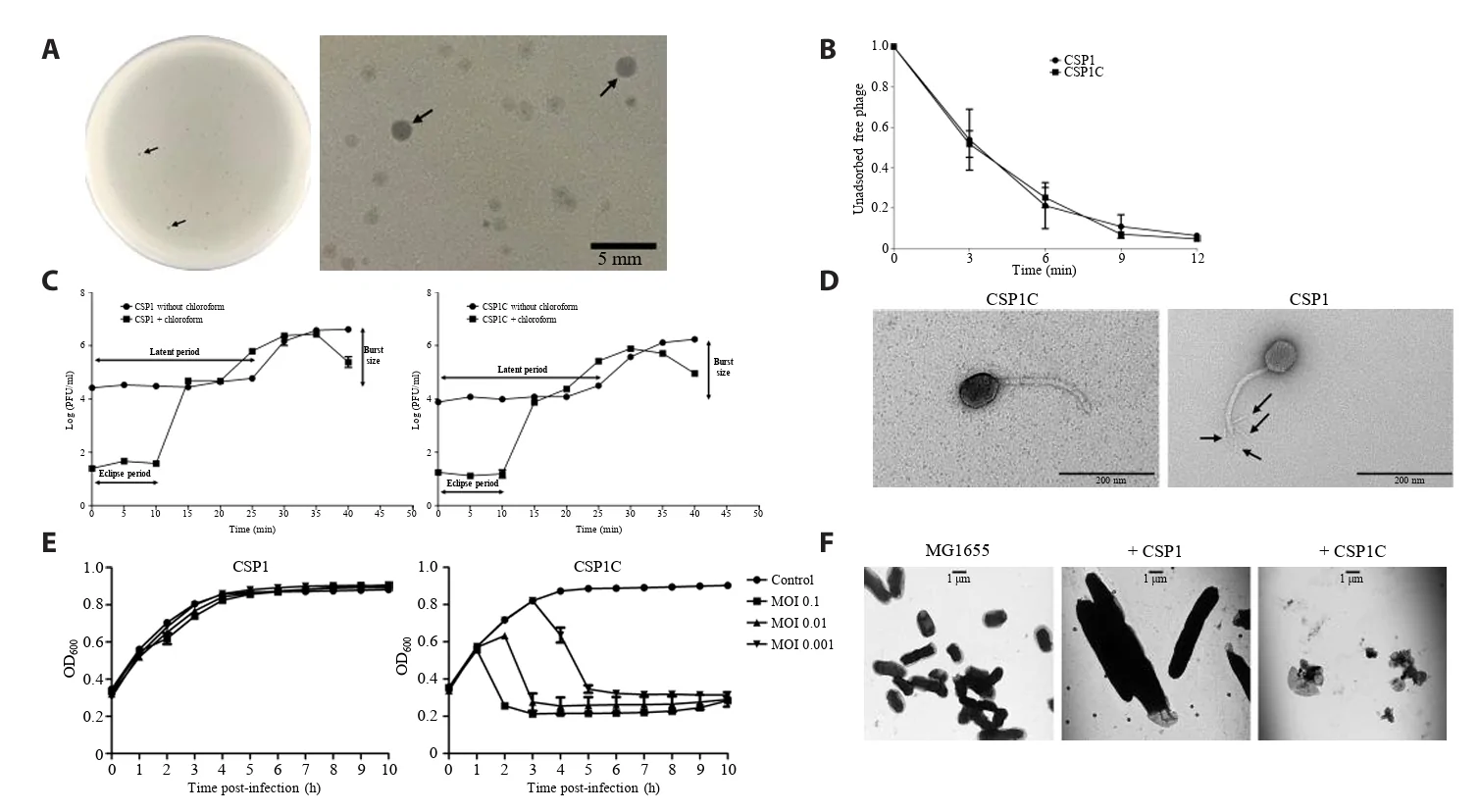
Phenotypic comparison of CSP1 and CSP1C. (A) Mixed plaques in CSP1 lysate, showing both turbid and clear plaques (left). CSP1 forms small turbid plaques, while CSP1C produces clear plaques (right). Black arrows indicate clear CSP1C plaques. (B) Adsorption rates of CSP1 and CSP1C in MG1655 at an MOI of 0.01. Adsorption rate is expressed as the ratio of unadsorbed free phages to the initial phage count after incubation. (C) One-step growth curve of CSP1 and CSP1C in MG1655, showing eclipse period, latent period and burst size. (D) TEM analysis of CSP1 and CSP1C. Black arrows indicate tail fibers. (E) Growth rate of MG1655 cultures after CSP1 or CSP1C infection at the specific MOIs. SM buffer was used as a control. (F) TEM analysis of MG1655 cells 2 h post-infection with CSP1 or CSP1C (MOI = 0.1).

CSP1 forms turbid plaques when infecting *E. coli* MG1655. However, distinct clear plaques were observed constantly even though the ratio was less than 0.01% of plaques formed (Fig. 1A). These clear plaques persisted after multiple rounds of single-plaque purification, suggesting that an irreversible spontaneous mutation in CSP1 may produce a clear plaque-forming variant. Previous analysis indicated that CSP1 lacks lysogeny-related genes, indicating that the observed low lytic activity is not due to lysogenization of its host (Kim et al., 2024). The small turbid plaques of CSP1 and clear plaques of CSP1C resemble the contrasting plaque morphologies observed in phage T4 and its lysis mutant r48. This similarity suggests that small turbid plaque formation by CSP1 might involve a LIN-like mechanism (Abedon, 2019). Although most plaques displayed distinct morphologies, the spontaneous emergence of CSP1C from CSP1 led to mixed phenotypes, indicating incomplete separation during plaque formation.

Although CSP1 and CSP1C exhibited different plaque morphologies, they showed comparable adsorption rates and replication dynamics. Approximately 90% of both phages adsorbed to the host cells in 12 min (Fig. 1B), and both phages had a latent period of 25 min (Fig. 1C). Their burst sizes were also nearly identical; CSP1 produced 172.42 ± 31.75 PFU/infected cell and CSP1C produced 168.94 ± 51.67 PFU/infected cell (Fig. 1C). TEM analysis showed that CSP1 possesses a tail fiber structure that is absent in CSP1C (n = 5) (Fig. 1D).

However, CSP1 and CSP1C showed differences in lytic activity. To compare the lytic activities, the turbidity of *E. coli* cultures was monitored at 1 h intervals up to 10 h by measuring OD_600_ following phage infection (Fig. 1E). The *E. coli* cultures were infected with phages at low MOIs of 0.1, 0.01, and 0.001 to minimize the effect of spontaneous generation of CSP1C during CSP1 infection. Reduction of culture turbidity was observed only in CSP1C-infected cultures, beginning at 1 h post-infection for an MOI of 0.1, 2 h for an MOI of 0.01, and 3 h for an MOI of 0.001. In contrast, turbidity was not reduced in a culture infected with CSP1 at the same MOIs. TEM analysis revealed that most cells were elongated without lysis 2 h after infection by CSP1, suggesting a possibility of delayed lysis by CSP1 (Fig. 1F). Quantitative analysis of cell length showed that the *E. coli* cells measured 2.08 ± 0.49 μm (n = 5), however, the elongated cells appeared after CSP1 infection had a length of 7.68 ± 2.18 μm (n = 5). Typical LIN of T4 is triggered by superinfection, which requires a high MOI. However, the lysis suppression in CSP1 was observed at low MOIs, suggesting a distinct mechanism of CSP1 LIN.

### Lytic activity of CSP1 and CSP1C

**Fig. 2.**
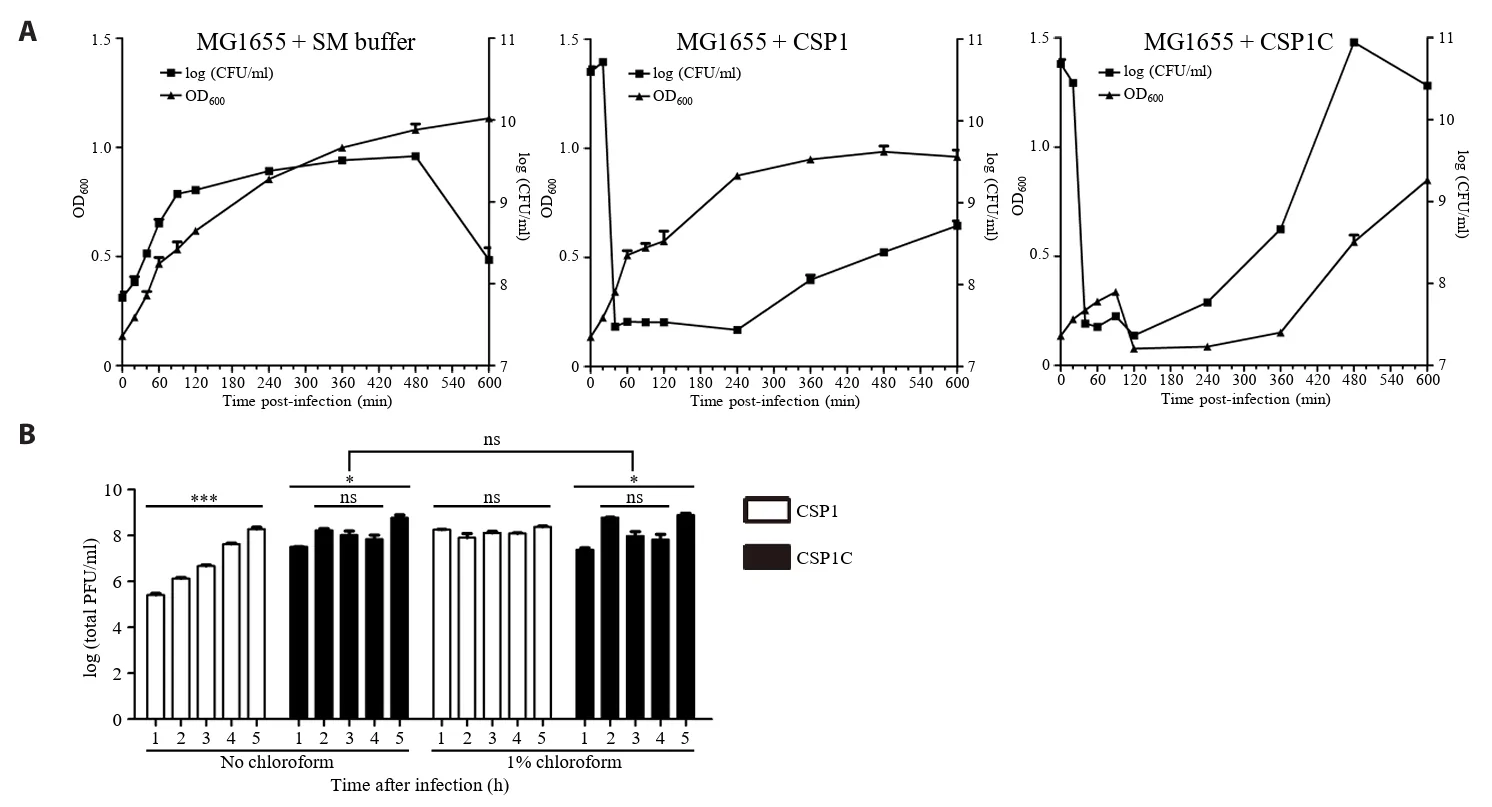
Lysis suppression in CSP1. (A) Growth inhibition (OD_600_) and reduction in colony-forming unit (CFU) of MG1655 cultures were measured after CSP1 or CSP1C infection at an MOI of 0.1. (B) PFU changes of CSP1 and CSP1C were monitored over 5 h incubation with MG1655 at an MOI of 0.1. Chloroform (1%) was added to release phage particles from unlysed cells. ^*^p<0.05; ^***^p<0.0001; ns, not significant.

A notable feature of typical LIN is an extended latent period, which retains cell viability to maximize progeny production (Abedon, 1992, 2009; Abedon et al., 2003). To analyze the lysis suppression caused by CSP1 infection, viable cell counts were measured after phage infection at an MOI of 0.1, which showed the greatest difference in turbidity reduction by CSP1 and CSP1C infections (Fig. 2A). Viable cell counts results indicated that CSP1 reduced viable cell number despite increased turbidity, suggesting a distinct LIN mechanism of CSP1 different from typical T4 LIN. Both phages exhibited a latent period of approximately 25 min (Fig. 1C) and caused host cell death. Lysis caused by CSP1C was observed at 90 min post-infection (Fig. 2A), suggesting that normal cell lysis occurs by CSP1C unlike CSP1.

The phage particles in the unlysed bacterial cells can be released by chloroform treatment (Abedon, 2019; Moussa et al., 2012). The concentration of CSP1 increased 100-fold upon chloroform treatment of *E. coli* cells at 1 h post-infection. However, the increase in CSP1C concentration was not observed (Fig. 2B). It is interesting to note that CSP1 displayed about 10-fold higher phage concentration compared to CSP1C after chloroform treatment despite having similar adsorption rates and replication dynamics (Fig. 1Band 1C). The higher phage concentration of CSP1 after chloroform treatment may be due to differences in the eclipse period. Some released CSP1C particles might enter an eclipse phase after adsorbing to neighboring bacteria. In contrast, most CSP1 particles remained within unlysed cells at 1 h post-infection and were not in an eclipse period. Similar increases in phage concentration after chloroform treatment were observed up to 4 h post-infection of CSP1. However, this increase was not detected for CSP1C, indicating that a large proportion of CSP1 particles are present in unlysed cells. Without chloroform treatment, CSP1 concentration gradually increased after 1 h post-infection, reaching levels similar to CSP1C at 5 h post-infection. In contrast, CSP1 concentration measured after chloroform treatment remained unchanged up to 5 h post-infection, indicating that CSP1 might not be amplified during lysis suppression. These results suggest that CSP1 employs a gradual and controlled release of phage particles over time, in contrast to the synchronized lysis seen in T4 (Abedon, 2019).

CSP1 and CSP1C had a latent period of 25 min, completing a replication cycle within 40 min (Fig. 1C). Both phages showed similar increases in turbidity and reductions in viable cell counts during this period (Fig. 2A). Additionally, CSP1 and CSP1C showed comparable PFU changes after chloroform treatment (Fig. 1C), indicating that lysis suppression was minimal during the first 40 min of infection.

Phages encounter challenging environments where bacterial populations are fluctuating under different conditions such as temperature and nutrient availability (Attrill et al., 2023; Engelhardt et al., 2014; Thomas et al., 2011; Williamson et al., 2013). In such conditions, the gradual release of CSP1 progeny likely prevents early depletion of susceptible hosts by maintaining a lower concentration of infectious particles. The moderate increase in the viable cell number observed 240 min post-CSP1 infection suggests that slow CSP1 release imposes lower selective pressure on the host population than CSP1C, allowing prolonged coexistence with susceptible hosts (Fig. 2A). This reduced selective pressure may be advantageous for prolonged survival of CSP1 compared to CSP1C, particularly in environments where hosts are scarce. These findings suggest that suppressed lysis in CSP1 may control infectious particle concentration, reducing early competition and supporting prolonged infection.

### Genomic comparison of CSP1 and CSP1C

**Fig. 3.**
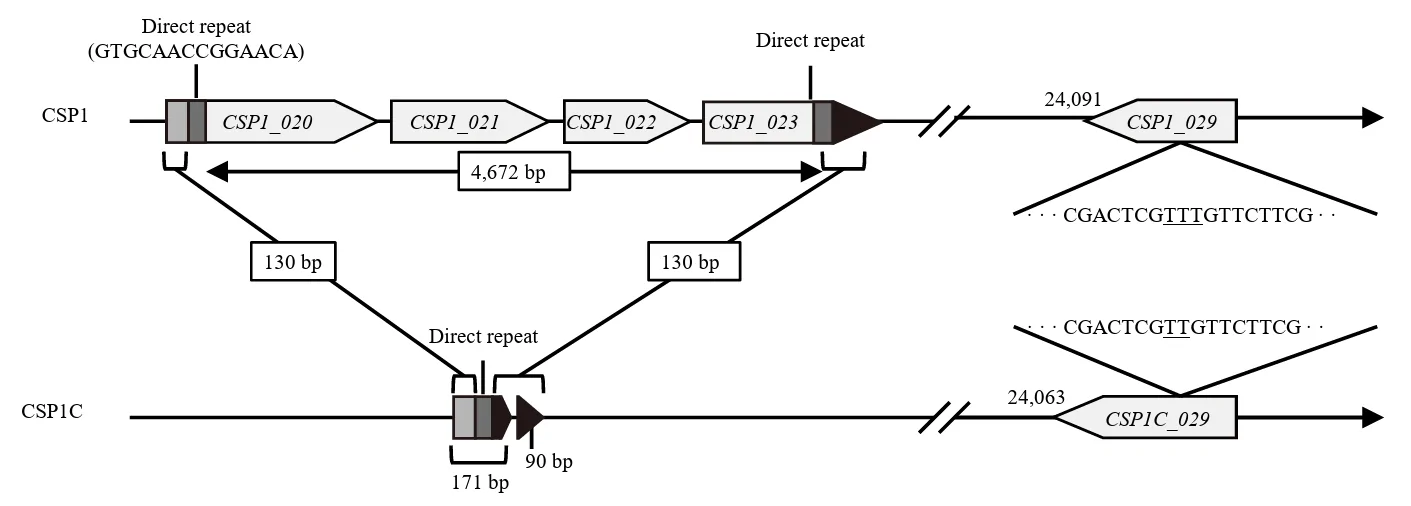
Schematic comparison of the mutated genes in CSP1 and CSP1C. The 4,672-bp deletion spans part of *CSP1_020*, *CSP1_021*, *CSP1_022*, and part of *CSP1_023*, which are flanked by 14-bp direct repeat sequences (GTGCAACCGGAACA). The deletion event left 130-bp fragments at both ends of *CSP1_020* and *CSP1_023*, resulting in two new ORFs (171 bp and 90 bp) in CSP1C. A single-nucleotide deletion in *CSP1_029* (underlined) shifts the translation termination site from 24,091 to 24,063.

The whole genome analysis of CSP1 and CSP1C revealed that CSP1 has a circular genome of 60,222 bp while CSP1C has a circular genome of 55,550 bp. Sequence alignment identified five-point mutations in addition to a deletion of 4,672 bp in CSP1C (Fig. 3). The five-point mutations include a deletion of T at nucleotide position 24,140, an addition of A at 49,132, and substitutions of G to A at 49,318, T to C at 52,083, and T to C at 60,173. Among these mutations, only the T deletion at 24,140 affects an ORF, *CSP1_029*. This mutation shifts its predicted translation termination site from position 24,091 to 24,063 without impacting the promoter or regulatory sequence of the downstream gene. The other point mutations occur in intergenic regions, sufficiently distant from promoters or regulatory sequences. Therefore, they are expected to have a minimal impact on the expression of neighboring genes (Table 1).

The deletion spans 4,672 bp from nucleotide positions 18,022 to 22,693 of CSP1, removing four ORFs: part of *CSP1_020* (3,081 bp), *CSP1_021* (708 bp), *CSP1_022* (369 bp), and part of *CSP1_023* (531 bp). This region is flanked by two 14-bp direct repeat sequences (GTGCAACCGGAACA), with one repeat sequence remaining in CSP1C after deletion. This implies that the deletion was probably caused by a DNA recombination or a DNA slippage event (Bi and Liu, 1996; Bzymek et al., 1999). Interestingly, DNA fragments of the same size, 130 bp from the 5′ end of *CSP1_020* and 130 bp from the 3′ end of *CSP1_023*, along with one remaining 14-bp direct repeat sequences, formed a 274-bp region in CSP1C. These sequences were predicted to create two new ORFs in CSP1C; CSP1C_020 (171 bp) and CSP1C_021 (90 bp). CSP1C_020 is predicted to encode a 56-amino-acid protein derived from the N-terminal region of a tail fiber protein CSP1_020, which consists of 1,026 amino acid residues. CSP1C_021 is predicted to encode a 29-amino-acid peptide derived from the C-terminal region of CSP1_023, which consists of 176 amino acid residues with an unknown function. CSP1_029 is predicted to encode a 59-amino-acid protein, with an unknown function. In CSP1C, a single deletion shifts the translation termination site of *CSP1_029*, resulting in a 68-amino-acid protein that retains the identical 44-amino-acid derived from the N-terminal region of CSP1_029. CSP1 and CSP1C displayed no differences in adsorption rate and replication dynamics, except for lytic activity, suggesting that these genes in CSP1C are likely non-functional.

Bioinformatic analysis of CSP1_020 revealed that its N-terminal region is homologous to a tail fiber protein, while its C-terminal region shares homology with a chaperone of endosialidase (Kim et al., 2024; Leiman et al., 2007). TEM images showed that CSP1 possesses a tail fiber structure, which is absent in CSP1C (Fig. 1D), confirming that *CSP1_020* encodes a tail fiber protein. This was further validated by the restoration of the tail fiber structure in CSP1C through *CSP1_020* complementation using *E. coli* expressing *CSP1_020* (Fig. S1). However, bioinformatic predictions could not reveal specific functions for CSP1_021, CSP1_022, CSP1_023, and CSP1_029.

### Identification of genes responsible for the suppressed lysis

**Fig. 4.**
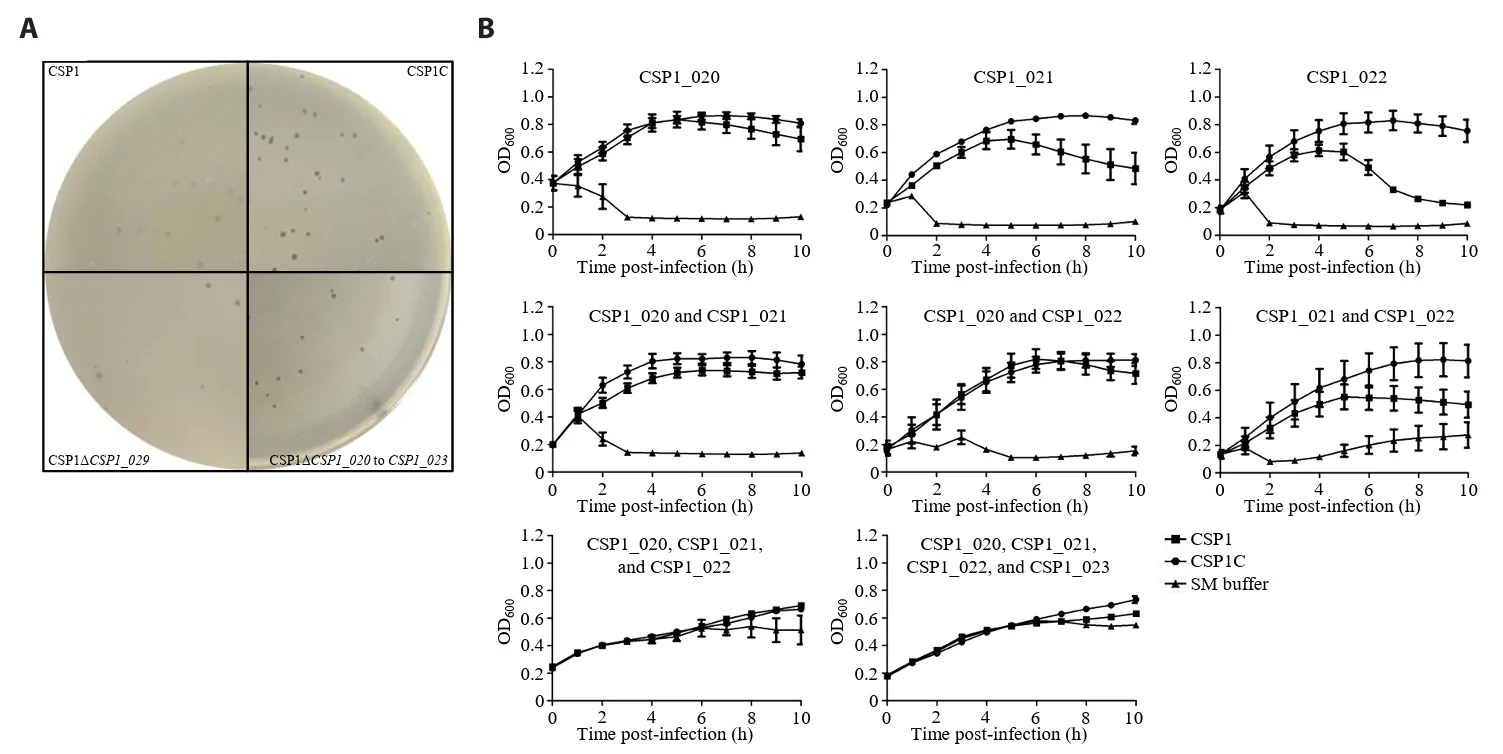
Identification of genes responsible for lysis suppression in CSP1. (A) Plaque morphology of CSP1, CSP1C, CSP1Δ*CSP1_029*, and CSP1Δ*CSP1_020* to *CSP1_023*. CSP1Δ*CSP1_020* to *CSP1_023* was constructed by deleting a 4,672-bp DNA region. (B) Complementation analysis of CSP1C with various combinations of *CSP1_020*, *CSP1_021*, *CSP1_022*, and *CSP1_023*. Growth rate of MG1655 expressing these gene combinations was monitored after CSP1C infection. Cultures were infected with CSP1 and CSP1C at an MOI of 0.1. SM buffer was used as a control. Detailed plasmid constructions are described in Table S2.

To identify the genes responsible for lysis suppression by CSP1, either the 4,672-bp DNA region or the *CSP1_029* was deleted from CSP1 using CRISPR-Cas9 system (Fig. 4A) (Hutinet et al., 2019; Jiang et al., 2013). Deletion of the 4,672-bp region in CSP1, which includes *CSP1_020*, *CSP1_021*, *CSP1_022*, and *CSP1_023* (Fig. 3), resulted in the formation of clear plaques. In contrast, deletion of *CSP1_029* did not alter the plaque turbidity of CSP1. These results suggest that *CSP1_029* is not involved in the lysis suppression, whereas genes within the 4,672-bp region may be associated with the lysis suppression in CSP1.

Infection of *E. coli* expressing the *CSP1_020*, *CSP1_021*, *CSP1_022*, and *CSP1_023* with CSP1C restored lysis suppression, suggesting that *CSP1_020*, *CSP1_021*, *CSP1_022*, and *CSP1_023* contribute to lysis suppression in CSP1 (Figs. 4B and S2). Systematic complementation analysis revealed that expressing a single gene or any combination of two genes among *CSP1_020*, *CSP1_021*, and *CSP1_022* in *E. coli* MG1655 did not restore the lysis suppression in CSP1C infection. However, simultaneous expression of all three genes fully restored the lysis suppression phenotype. These results suggest that *CSP1_020*, *CSP1_021*, and *CSP1_022* are required together to induce lysis suppression in CSP1C.

CSP1 contains three lysis-related genes; endolysin (*CSP1_091*), holin (*CSP1_092*), and spanin (*CSP1_093*), which constitute the holin-endolysin system responsible for lysing Gram-negative host cells. The cell elongation observed in *E. coli* infected with CSP1 suggests that lysis suppression occurs before spanin activity. Blocking spanin typically results in spheroid-shaped cells due to the peptidoglycan loss (Fig. 1F) (Cahill et al., 2024; Rajaure et al., 2015). This observation rules out spanin inhibition as the cause of lysis suppression by CSP1. These findings suggest that CSP1_020, CSP1_021, and CSP1_022 may inhibit endolysin function, either by directly acting on endolysin or by modulating holin activity. A study on the LambdaSo phage demonstrated that holin mutations, which prevent proper lysis, can result in extensive bacterial elongation (Thöneböhn et al., 2024). Since CSP1 lysis suppression is mediated by antiholin activity, this activity may contribute to elongation. However, whether cell elongation is an intrinsic consequence of lysis suppression or simply a byproduct of delayed lysis remains unclear. CSP1 lysis suppression prevents rapid host cell lysis, allowing infected cells to remain unlysed for an extended period, which may facilitate elongation. In contrast, CSP1C-infected cells undergo rapid lysis that cell elongation cannot be observed.

CSP1_020 is predicted to be a tail fiber protein with depolymerase activity. Phage-encoded depolymerases, such as endosialidase, degrade host cell polysaccharides to improve access to bacterial receptors. This process generates halos around plaques without affecting plaque turbidity (Abdelkader et al., 2022; Knecht et al., 2020; Leprince and Mahillon, 2023; Wu et al., 2019). CSP1 encodes two receptor-binding proteins, CSP1_018 and CSP1_020, and utilizes LamB as a receptor for *E. coli* MG1655 infection (Kim et al., 2024). CSP1_018 has high homology to protein J of phage lambda that it is expected to bind LamB. CSP1C lost CSP1_020, which is unlikely to affect CSP1C adsorption and plaque turbidity on *E. coli*, even though it is essential for lysis suppression in CSP1. CSP1_021 and CSP1_022 were predicted to be proteins with unknown functions. Therefore, their possible cellular localizations were explored by predicting transmembrane domain. A transmembrane domain was identified in CSP1_021, whereas none was found in CSP1_022. This suggests that CSP1_021 is localized in the membrane and may interact with holin.

### Antiholin activity in CSP1

**Fig. 5.**
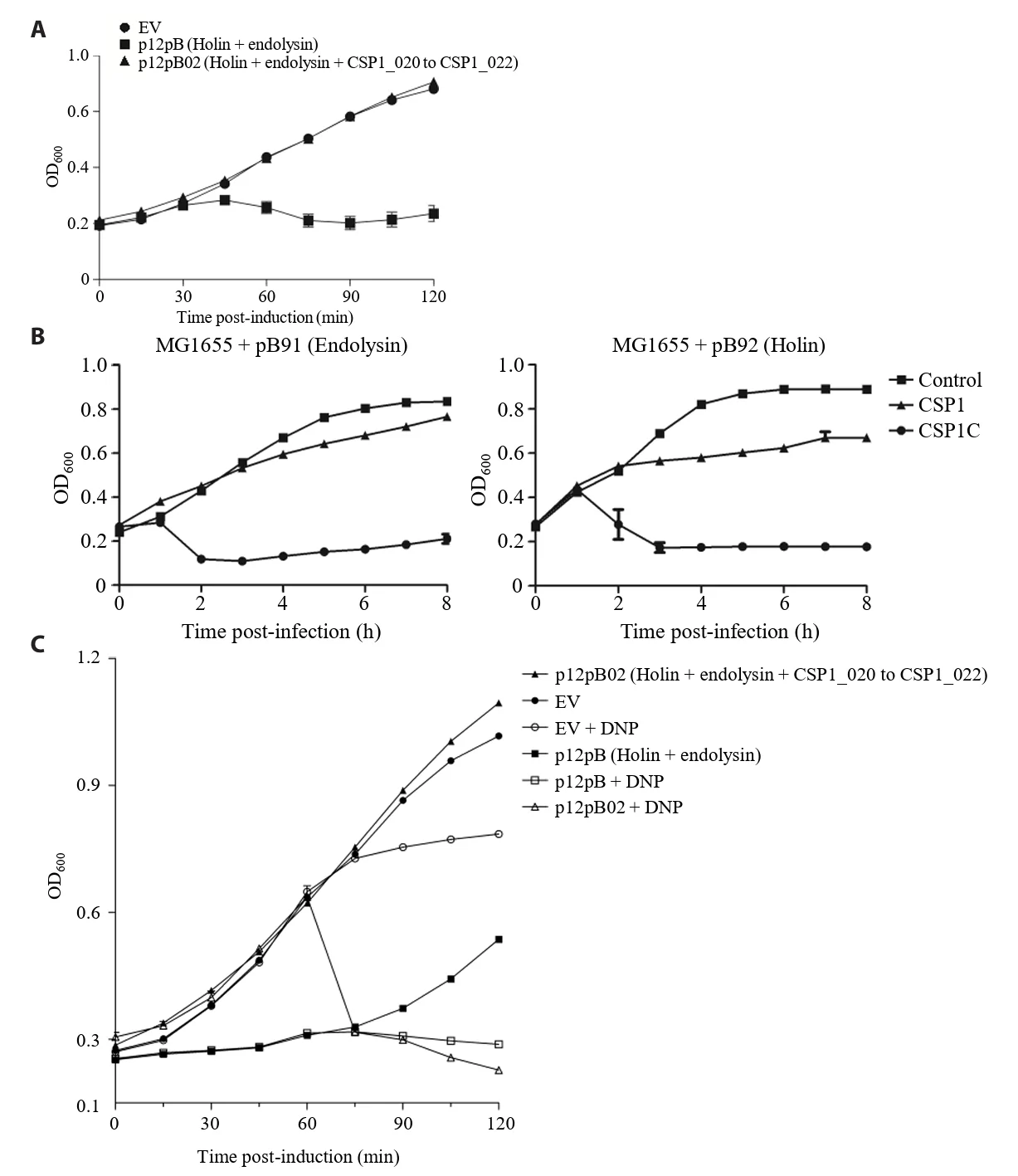
Antiholin activity of CSP1_020, CSP1_021, and CSP1_022. (A) Growth rate of BL21 (DE3) co-expressing *CSP1_020*, *CSP1_021*, and *CSP1_022* with holin and endolysin. Expression of both holin and endolysin (p12) was induced with 0.2 mM IPTG, and expression of CSP1_020, CSP1_021, and CSP1_022 (pB02) was induced with 0.2% L-arabinose. (B) Growth rate of MG1655 expressing either endolysin (left) or holin (right) after CSP1 infection. The protein expressions were induced with 0.2% L-arabinose. SM buffer was used as a control. (C) Growth rate of BL21 (DE3) co-expressing endolysin, holin, and CSP1_020, CSP1_021, and CSP1_022 in the presence or absence of 2 mM DNP. DNP was added 1 h post-induction to disrupt the PMF, and OD_600_ was monitored for 120 min. Holin and endolysin expression was induced by 0.2 mM IPTG, and CSP1_020, CSP1_021, and CSP1_022 expression was induced by 0.2% L-arabinose. EV: empty vector

The possible role of CSP1_020, CSP1_021, and CSP1_022 in lysis suppression through inhibition of the holin-endolysin system was further examined by co-expressing CSP1_020, CSP1_021, and CSP1_022 with holin and endolysin in *E. coli* BL21 (DE3) (Fig. 5A). This co-expression effectively led to lysis suppression, demonstrating that CSP1_020, CSP1_021, and CSP1_022 function together to control lysis by modulating the holin-endolysin system. To determine whether CSP1 lysis suppression resulted from endolysin or holin inhibition, *E. coli* MG1655 overexpressing either endolysin or holin was infected with CSP1. Overexpression of endolysin or holin was expected to overcome the CSP1-mediated lysis suppression (Fig. 5B). Lysis suppression was maintained in the strain overexpressing endolysin. However, it was disrupted in the strain overexpressing holin, suggesting that lysis suppression by CSP1 is mediated by antiholin activity. LIN in coliphages, mediated by antiholin activity, can be disrupted by PMF collapse, which triggers holin pore formation (Abedon, 1992; Hays and Seed, 2020; Heagy, 1950). To further confirm the antiholin function of CSP1_020, CSP1_021, and CSP1_022, lysis-suppressed *E. coli* BL21 (DE3) was exposed to 2,4-dinitrophenol (DNP), an ionophore that disrupts the PMF (Fig. 5C) (Hays and Seed, 2020). DNP treatment induced rapid lysis of cells in which lysis suppression was established by co-expression of CSP1_020, CSP1_021, and CSP1_022 with holin and endolysin, further supporting their antiholin activity.

**Fig. 6.**
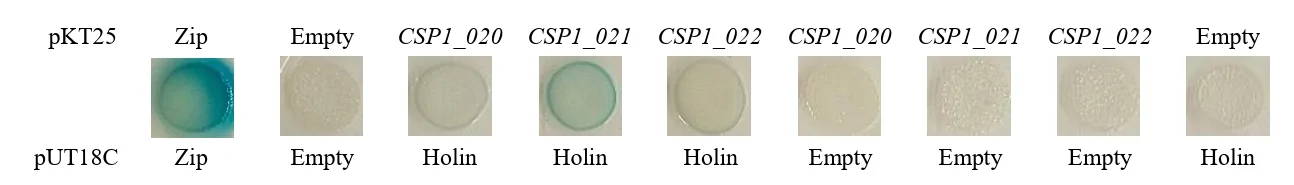
Blue signals indicate positive interactions. The top row represents genes cloned in the pKT25 vector, while the bottom row represents holin cloned in the pUT18C vector. Zip was used as a positive control, and Empty indicates an empty vector as a negative control.

A bacterial two-hybrid analysis was performed to investigate interactions between CSP1_020, CSP1_021, CSP1_022, and holin (Fig. 6). The results showed that CSP1_021 produced a blue signal with holin, indicating a direct interaction and supporting its function as an antiholin. In contrast, CSP1_020 and CSP1_022 showed no detectable signals, suggesting that they may not directly interact with holin. Instead, they may contribute to lysis suppression in CSP1 through mechanisms distinct from direct antiholin activity. In T4 phages, the antiholin activity of RI and RIII proteins is the primary pathway for LIN. However, additional factors, such as the poorly characterized RV protein, are also thought to influence infection efficiency and may contribute to LIN (Dressman and Drake, 1999; Slavcev and Hayes, 2003). Similarly, CSP1 may employ multiple mechanisms for lysis suppression, combining direct antiholin activity via CSP1_021 with unknown roles of CSP1_020 and CSP1_022.

This study provides insights into the novel lysis suppression in CSP1 through a comparative analysis with its mutant phage, CSP1C. We identified three novel proteins, CSP1_020, CSP1_021, and CSP1_022, that control lysis suppression in CSP1 via antiholin activity. Unlike T4 antiholin proteins, RI and RIII, which independently exhibit antiholin activity (Chen and Young, 2016), all three CSP1 proteins are required together for antiholin function. RI protein contains a single transmembrane domain and is released into the periplasm via its signal anchor domain (Tran et al., 2007). Similarly, CSP1_021 is predicted to contain a single transmembrane domain. However, it lacks a signal peptide, indicating it remains anchored to the inner membrane rather than being released into the periplasm. CSP1_092 is predicted as a class I holin with three transmembrane domains capable of forming large membrane pores to induce cell lysis. The interaction between CSP1_021 and CSP1_092 resembles the system observed in phage P1, where the single transmembrane domain antiholin LydB interacts with the class I holin LydA (Bednarek et al., 2022; Young and White, 2008). Antiholin expression is typically co-regulated with lysis genes in a temperate phage (Guo et al., 2018). However, *CSP1_020*, *CSP1_021*, and *CSP1_022* genes are located separately from the *CSP1_092*, suggesting that CSP1 regulates lysis differently from temperate phages.

Our observations indicate that CSP1 lysis suppression reduces bacterial viability without increasing progeny production (Figs. 1C, 2A, 2B), distinguishing it from the LIN in T4. Lysis suppression in CSP1 occurs even at low MOIs by slowing phage release, potentially reducing the rate of host depletion. The spontaneous emergence of CSP1C, a lytic mutant, during CSP1 infection further highlights the unique dynamics between these two phages. CSP1 lysis suppression reduces CSP1C release and mitigates the rapid host depletion caused by strong lytic activity of CSP1C. This prolongs infection and helps to balance phage-host dynamics over time. The new LIN mechanism discovered in CSP1 can enhance our knowledge about lysis regulation in phage-host interactions. Future studies should clarify the individual roles and interactions of CSP1_020, CSP1_021, and CSP1_022, particularly their mechanism of holin inhibition.

## Figures and Tables

**Table 1.** List of mutated genes in CSP1

Gene	Product	Mutation
*CSP1_020*	Tail fiber protein	2,951 bp from 3′ end deletion
*CSP1_021*	Hypothetical protein	Gene knockout
*CSP1_022*	Hypothetical protein	
*CSP1_023*	Hypothetical protein	514 bp from 5′ end deletion
*CSP1_029*	Hypothetical protein	24,140T deletion
Intergenic region	None	49,132A addition
		49,318G to A substitution
		52,083T to C substitution
		60,173T to C substitution

## Data Availability

The complete genome sequence of phage CSP1 and CSP1C has been deposited in GenBank under accession numbers KY982929 and PQ20298. Further inquiries can be directed to the corresponding author.

## Supplementary Information

Table S1. Plasmids used in this study

Table S2. Bacterial strains used in this study

Table S3. Oligonucleotide sequences for spacers

Table S4. Oligonucleotide sequences for repair plasmids

Table S5. Primers used for constructing complementation plasmids for CSP1C

Table S6. Primers used for constructing endolysin and holin expression plasmids

Table S7. Primers used for constructing plasmids for antiholin activity test

Table S8. Primers used for constructing plasmids for bacterial two-hybrid assay

Fig. S1. TEM images of (A) CSP1C produced in *E. coli* MG1655 and (B) CSP1C produced in *E. coli* MG1655 harboring pACYC184::*CSP1_020* for *CSP1_020* complementation.

Fig. S2. Lytic activity of CSP1 and CSP1C against MG1655 harboring empty vectors. Cultures were infected with CSP1 and CSP1C at an MOI of 0.1. SM buffer was used as a control. pApU (pACYC184 + pUHE21-2 *lacI^q^*) and pBpU (pBAD33 + pUHE21-2 *lacI^q^*) represent empty vectors controls.
