# Supplementary material for: Genetic insights into novel lysis suppression by phage CSP1 in Escherichia coli
Source: J Microbiol. 2025 Apr 29;63(4):e2501013. doi: 10.71150/jm.2501013 (PMC13577005; doi:10.71150/jm.2501013)
Supplement: Supplementary file 1 [file jm-2501013-Supplementary-Fig-S1.docx]

**Fig. S1.** TEM images of (A) CSP1C produced in *E. coli* MG1655 and (B) CSP1C produced in *E. coli* MG1655 harboring pACYC184::*CSP1_020* for *CSP1_020* complementation.
