# Supplementary material for: Genetic insights into novel lysis suppression by phage CSP1 in Escherichia coli
Source: J Microbiol. 2025 Apr 29;63(4):e2501013. doi: 10.71150/jm.2501013 (PMC13577005; doi:10.71150/jm.2501013)
Supplement: Supplementary file 2 [file jm-2501013-Supplementary-Fig-S2.docx]

**Fig. S2.** Lytic activity of CSP1 and CSP1C against MG1655 harboring empty vectors. Cultures were infected with CSP1 and CSP1C at an MOI of 0.1. SM buffer was used as a control. pApU (pACYC184 + pUHE21-2 *lacI^q^*) and pBpU (pBAD33 + pUHE21-2 *lacI^q^*) represent empty vectors controls.
