# Supplementary material for: Genetic insights into novel lysis suppression by phage CSP1 in Escherichia coli
Source: J Microbiol. 2025 Apr 29;63(4):e2501013. doi: 10.71150/jm.2501013 (PMC13577005; doi:10.71150/jm.2501013)
Supplement: Supplementary file 3 [file jm-2501013-Supplementary-Table-S1.docx]

**Table S1**. Plasmids used in this study

| **Plasmid** | **Description** | **Reference** |
| --- | --- | --- |
| pACYC184 (pA) | Low copy uninducible vector. Cm^R^ | Mok et al. (1991) |
| pBAD33 (pB) | Arabinose inducible vector. Cb^R^ | Guzman et al. (1995) |
| pUHE21-2 *lacI^q^* (pU) | Lactose inducible vector with *lacI^q^* repressor. Cb^R^ | Soncini et al. (1995) |
| pETDuet-1 | Lactose inducible vector with *lacI*. Two MCS sites, each with its own T7 promoters. Cb^R^ | Lab. collection |
| pA0pU | pACYC184 expressing *CSP1_020* and empty pUHE21-2 *lacI^q^* | This study |
| pB1pU | pACYC184 expressing *CSP1_021* and empty pUHE21-2 *lacI^q^* | This study |
| pBpU2 | pUHE21-2 *lacI^q^* expressing *CSP1_022* and empty pBAD33 | This study |
| pA01pU | pACYC184 expressing *CSP1_020* to *CSP1_021* and empty pUHE21-2 *lacI^q^* | This study |
| pA0pU2 | pACYC184 expressing *CSP1_*020 and pUHE21-2 *lacI^q^* expressing CSP1*_022* | This study |
| pB1pU2 | pBAD33 expressing *CSP1_021* and pUHE21-2 *lacI^q^* expressing CSP1*_022* | This study |
| pB91 | pBAD33 expressing endolysin gene, *CSP1_091* | This study |
| pB92 | pBAD33 expressing holin gene, *CSP1_092* | This study |
| P | pETDuet-1 | This study |
| p12 | pETDuet-1 expressing endolysin at MCS1 and holin at MCS2 | This study |
| pB02 | pBAD33 expressing CSP1_020 to CSP1_022 | This study |
| pKT25 | Amp^R^, plasmid for bacterial two-hybrid assay | Karimova et al. (2001) |
| pUT18C | Km^R^, plasmid for bacterial two-hybrid assay | Karimova et al. (2001) |
| pKT25-Zip | positive control for bacterial two-hybrid assay | Karimova et al. (2001) |
| pUT18C-Zip | positive control for bacterial two-hybrid assay | Karimova et al. (2001) |
| pKT25::*CSP1_020* | pKT25 expressing CSP1_020 | This study |
| pKT25::*CSP1_021* | pKT25 expressing CSP1_021 | This study |
| pKT25::*CSP1_022* | pKT25 expressing CSP1_022 | This study |
| pUT18C::Holin | pKT25 expressing holin (CSP1_092) | This study |
