# Supplementary material for: Genetic insights into novel lysis suppression by phage CSP1 in Escherichia coli
Source: J Microbiol. 2025 Apr 29;63(4):e2501013. doi: 10.71150/jm.2501013 (PMC13577005; doi:10.71150/jm.2501013)
Supplement: Supplementary file 4 [file jm-2501013-Supplementary-Table-S2.docx]

**Table S2.** Bacterial strains used in this study

| **Strains** | **Genotype** | **Source** |
| --- | --- | --- |
| *Escherichia coli* | | |
| MG1655 | WT | Lab. strain |
| MG1655 + pApU | WT + pACYC184 + pUHE21-2 *lacI^q^* | This study |
| MG1655 + pA03pU | WT + pACTC184::*CSP1_020 ~ CSP1_023* + pUHE21-2 *lacI^q^* | This study |
| MG1655 + pA02pU | WT + pACTC184::*CSP1_020 ~ CSP1_022* + pUHE21-2 *lacI^q^* | This study |
| MG1655 + pA01pU | WT + pACTC184::*CSP1_020 ~ CSP1_021* + pUHE21-2 *lacI^q^* | This study |
| MG1655 + pA0pU | WT + pACTC184::*CSP1_020* + pUHE21-2 *lacI^q^* | This study |
| MG1655 + pA1pU | WT + pACTC184::*CSP1_021* + pUHE21-2 *lacI^q^* | This study |
| MG1655 + pApU2 | WT + pACTC184 + pUHE21-2 *lacI^q^*::*CSP1_022* | This study |
| MG1655 + pA01pU | WT + pACTC184::*CSP1_020 ~ CSP1_021* + pUHE21-2 *lacI^q^* | This study |
| MG1655 + pA0pU2 | WT + pACTC184::*CSP1_020* + pUHE21-2 *lacI^q^*::*CSP1_022* | This study |
| MG1655 + pA1pU2 | WT + pACTC184::*CSP1_021*+ pUHE21-2 *lacI^q^*::*CSP1_022* | This study |
| MG1655 + pB91 | WT + pBAD33::endolysin | This study |
| MG1655 + pB92 | WT + pBAD33::holin | This study |
| BL21 (DE3) + ppB | WT + pETDuet-1 + pBAD33 | This study |
| BL21 (DE3) + p12pB | WT + pETDuet-1::*CSP1_091*, *CSP1_092* + pBAD33 | This study |
| BL21 (DE3) + p12pB02 | WT + pETDuet-1::*CSP1_091*, *CSP1_092* + pBAD33::*CSP1_020* to *CSP1_022* | This study |
| DH5a | WT | This study |
| BTH101 + pUT18C + pKT25 | WT + pUT18C + pKT25 | This study |
| BTH101 + pUT18C::Holin + pKT25 | WT + pUT18C::Holin + pKT25 | This study |
| BTH101 + pUT18C::Holin + pKT25::*CSP1_020* | WT + pUT18C::Holin + pKT25::*CSP1_020* | This study |
| BTH101 + pUT18C::Holin + pKT25::*CSP1_021* | WT + pUT18C::Holin + pKT25::*CSP1_021* | This study |
| BTH101 + pUT18C::Holin + pKT25::*CSP1_022* | WT + pUT18C::Holin + pKT25::*CSP1_022* | This study |
| BTH101 + pUT18C + pKT25::*CSP1_020* | WT + pUT18C + pKT25::*CSP1_020* | This study |
| BTH101 + pUT18C + pKT25::*CSP1_021* | WT + pUT18C + pKT25::*CSP1_021* | This study |
| BTH101 + pUT18C + pKT25::*CSP1_022* | WT + pUT18C + pKT25::*CSP1_022* | This study |
| BTH101 + pUT18C-Zip + pKT25-Zip | WT + pUT18C-Zip + pKT25-Zip | This study |
