# Supplementary material for: Genetic insights into novel lysis suppression by phage CSP1 in Escherichia coli
Source: J Microbiol. 2025 Apr 29;63(4):e2501013. doi: 10.71150/jm.2501013 (PMC13577005; doi:10.71150/jm.2501013)
Supplement: Supplementary file 5 [file jm-2501013-Supplementary-Table-S3.docx]

**Table S3.** Oligonucleotide sequences for spacers

| **Primers** | | **Spacer Sequence (5′ to 3′)** |
| --- | --- | --- |
| 4.6 kb deletion | Forward | AAACACAGAAGCTTGCACTTCCACG |
|  | Reverse | AAAACGTGGAAGTGCAAGCTTCTGT |
| *CSP1_029* deletion | Forward | AAACGAGCAAACAAGAAGCGTTAAG |
|  | Reverse | AAAACTTAACGCTTCTTGTTTGCTC |
