# Supplementary material for: Genetic insights into novel lysis suppression by phage CSP1 in Escherichia coli
Source: J Microbiol. 2025 Apr 29;63(4):e2501013. doi: 10.71150/jm.2501013 (PMC13577005; doi:10.71150/jm.2501013)
Supplement: Supplementary file 6 [file jm-2501013-Supplementary-Table-S4.docx]

**Table S4.** Oligonucleotide sequences for repair plasmids

| **Primers** | | **Sequence (5′ to 3′)** |
| --- | --- | --- |
| 4.6 kb  Deletion  Fragment 1 | Forward | GTTGTAAAACGACGGCCAGTGGACTGCCTAAACAGCAACGGTTAGTATTACCAC |
|  | Reverse | GCTATGACCATGATTACGCCATCAATCTCATGGCACCATTGTCCAGTCATTGG |
| *CSP1_029*  deletion  Fragment 1 | Forward | GCTATGACCATGATTACGCCTGTCTGTAACCTTTTGCTGC |
|  | Reverse | CCTTAAAGGGGTATATTAGCGCCAACTCCGGAATCACCGG |
| *CSP1_029* deletion  Fragment 1 | Forward | CCGGTGATTCCGGAGTTGGCGCTAATATACCCCTTTAAGG |
|  | Reverse | GTTGTAAAACGACGGCCAGTAAAAGAACGCACGTTGGCTG |
| Gibson assembly  (Vector Fragment 1) | Forward | TACAGGCATCGTGGTGTCACGCTCGTCGTTTGGTATGGCTT |
|  | Reverse | GGCGTAATCATGGTCATAGCTGTTTCCTGT |
| Gibson assembly  (Vector Fragment 2) | Forward | ACTGGCCGTCGTTTTACAACGTCGTGACTG |
|  | Reverse | AAGCCATACCAAACGACGAGCGTGACACCACGATGCCTGTA |
