# Supplementary material for: Genetic insights into novel lysis suppression by phage CSP1 in Escherichia coli
Source: J Microbiol. 2025 Apr 29;63(4):e2501013. doi: 10.71150/jm.2501013 (PMC13577005; doi:10.71150/jm.2501013)
Supplement: Supplementary file 7 [file jm-2501013-Supplementary-Table-S5.docx]

**Table S5.** Primers used for constructing complementation plasmids for CSP1C

| **Primers** | | **Sequence (5′ to 3′)** |
| --- | --- | --- |
| *CSP1_020* | Forward | ATAGGATCCTTTTGTGTTGTTGTCAAGGTTCAATTCTAA |
|  | Reverse | ATAGTCGACATGGATTTGTTAAGCGGATGTAACCAACTG |
| *CSP1_021* | Forward | ATAGGATCCATGTCTGACATGACTCTTTTACCTACTAAT |
|  | Reverse | ATAGTCGACCCACACTTCAACGATTAGCAGCAGCCAGTC |
| *CSP1_022* | Forward | ATAGGATCCATGAGCCACATTAAATTCGCAACGATGGGA |
|  | Reverse | ATAGTCGACGTCGGCTTCCTATTCTTACATATCTTTCAT |
| *CSP1_023* | Forward | ATAGGATCCGAATAGGAAGCCGACATGCAAAACGTAAAC |
|  | Reverse | ATAGTCGACAGAAAGTCCTTCAACAACTTTATCAATACC |
