# Supplementary material for: Genetic insights into novel lysis suppression by phage CSP1 in Escherichia coli
Source: J Microbiol. 2025 Apr 29;63(4):e2501013. doi: 10.71150/jm.2501013 (PMC13577005; doi:10.71150/jm.2501013)
Supplement: Supplementary file 8 [file jm-2501013-Supplementary-Table-S6.docx]

**Table S6.** Primers used for constructing endolysin and holin expression plasmids

| **Primers** | | **Sequence (5′ to 3′)** |
| --- | --- | --- |
| Endolysin | Forward | ATAGGATCCATGGCACTTCAACCTCGTGGTATTCGTAAT |
|  | Reverse | ATAGTCGACTCCCTCCAATAAAAAGGGGCTGACGCC |
| Holin | Forward | ATAGGATCCCCTTTTTATTGGAGGGAACATG |
|  | Reverse | ATAAAGCTTCATCAGGTACTCGATAAATAGACGC |
