# Supplementary material for: Genetic insights into novel lysis suppression by phage CSP1 in Escherichia coli
Source: J Microbiol. 2025 Apr 29;63(4):e2501013. doi: 10.71150/jm.2501013 (PMC13577005; doi:10.71150/jm.2501013)
Supplement: Supplementary file 9 [file jm-2501013-Supplementary-Table-S7.docx]

**Table S7.** Primers used for constructing plasmids for antiholin activity test

| **Vector** | **Primer** | | **Sequence (5′ to 3′)** |
| --- | --- | --- | --- |
| pETDuet-1 | Endolysin | Forward | ATAGGATCCATGGCACTTCAACCTCGTGGTATTCGTAAT |
|  |  | Reverse | ATAGTCGACTCCCTCCAATAAAAAGGGGCTGACGCC |
|  | Holin | Forward | ATATCTAGATTCAACTCATCAAACGAACATTGGGCAGTT |
|  |  | Reverse | ATAGGTACCCATCAGGTACTCGATAAATAGACGC |
| pBAD33 | *CSP1_020* to *CSP1_022* | Forward | ATAGGATCCTTTTGTGTTGTTGTCAAGGTTCAATTCTAA |
|  |  | Reverse | ATAGTCGACGTCGGCTTCCTATTCTTACATATCTTTCAT |
