# Supplementary material for: Genetic insights into novel lysis suppression by phage CSP1 in Escherichia coli
Source: J Microbiol. 2025 Apr 29;63(4):e2501013. doi: 10.71150/jm.2501013 (PMC13577005; doi:10.71150/jm.2501013)
Supplement: Supplementary file 10 [file jm-2501013-Supplementary-Table-S8.docx]

**Table S8.** Primers used for constructing plasmids for bacterial two-hybrid assay

| **Vector** | **Primer** | | **Sequence (5′ to 3′)** |
| --- | --- | --- | --- |
| pUT18C | Holin (CSP1_092) | Forward | ATATCTAGATTCAACTCATCAAACGAACATTGGGCAGTT |
|  |  | Reverse | ATAGGTACCCATCAGGTACTCGATAAATAGACGC |
| pKT25 | *CSP1_020* | Forward | ATATCTAGAGCAACTCGTTTACGTGGTACTTTAGTTG |
|  |  | Reverse | ATAGGTACCTGTTGAAGTTAGATGTGGTTATGGTAATGG |
|  | *CSP1_021* | Forward | ATATCTAGATCTGACATGACTCTTTTACCTACTAATCTCG |
|  |  | Reverse | ATAGGTACCCCACACTTCAACGATTAGCAGCAGCCAGTC |
|  | *CSP1_022* | Forward | ATATCTAGAAGCCACATTAAATTCGCAACGATGGGAATG |
|  |  | Reverse | ATAGGTACCGTCGGCTTCCTATTCTTACATATCTTTCAT |

**References**

Guzman LM, Belin D, Carson MJ, Beckwith J. 1995. Tight regulation, modulation, and high-level expression by vectors containing the arabinose PBAD promoter. *J Bacteriol*. 177(14): 4121–4130.

Karimova G, Ullmann A, Ladant D. 2001. Protein-protein interaction between *Bacillus stearothermophilus* tyrosyl-tRNA synthetase subdomains revealed by a bacterial two-hybrid system. *J Mol Microbiol Biotechnol*. 3(1): 73–82.

Mok YK, Clark DR, Kam KM, Shaw PC. 1991. *Bsi* Y I, a novel thermophilic restriction endonuclease that recognizes 5′ CCNNNNNNNGG 3′ and the discovery of a wrongly sequenced site in pACYC177. *Nucleic Acids Res*. 19(9): 2321–2323.

Soncini FC, Véscovi EG, Groisman EA. 1995. Transcriptional autoregulation of the *Salmonella typhimurium* *phoPQ* operon. *J Bacteriol*. 177(15): 4364–4371.
